# Similarities and differences in the *functional architecture* of mother- infant communication in rhesus macaque and British mother-infant dyads

**DOI:** 10.1038/s41598-023-39623-3

**Published:** 2023-08-13

**Authors:** V. Sclafani, L. De Pascalis, L. Bozicevic, A. Sepe, P. F. Ferrari, L. Murray

**Affiliations:** 1 Winnicott Research Unit, Department of Psychology, University of Reading, Reading, UK; 2 College of Social Sciences, School of Psychology, University of Lincoln, Brayford Pool, Lincoln, LN6 7TS UK; 3 Department of Psychology, Institute of Population Health, University of Liverpool, Liverpool, UK; 4 Department of Psychology, University of Bologna, Bologna, Italy; 5 Department of Primary Care & Mental Health, Institute of Population Health, University of Liverpool, Liverpool, Merseyside UK; 6 Department of Medicine and Surgery, University of Parma, Parma, Italy; 7 Laboratory of Neuro- and Psychophysiology, Department of Neurosciences, KU Leuven Medical School, Leuven, Belgium; 8 Institut des Sciences Cognitives ‘Marc Jeannerod’, CNRS, Bron, and Université Claude Bernard Lyon 1, Lyon, France

**Keywords:** Evolution, Psychology

## Abstract

Similarly to humans, rhesus macaques engage in mother-infant face-to-face interactions. However, no previous studies have described the naturally occurring structure and development of mother-infant interactions in this population and used a comparative-developmental perspective to directly compare them to the ones reported in humans. Here, we investigate the development of infant communication, and maternal responsiveness in the two groups. We video-recorded mother-infant interactions in both groups in naturalistic settings and analysed them with the same micro-analytic coding scheme. Results show that infant social expressiveness and maternal responsiveness are similarly structured in humans and macaques. Both human and macaque mothers use specific mirroring responses to specific infant social behaviours (modified mirroring to communicative signals, enriched mirroring to affiliative gestures). However, important differences were identified in the development of infant social expressiveness, and in forms of maternal responsiveness, with vocal responses and marking behaviours being predominantly human. Results indicate a common *functional architecture* of mother-infant communication in humans and monkeys, and contribute to theories concerning the evolution of specific traits of human behaviour.

## Introduction

Mutually responsive face-to-face interactions between human parents and their infants from around two–three months postpartum have been well-described in the psychological literature, since the seminal descriptions in the 1970’s^1–6^, and play an important role in the development of infant cognitive and emotional development^7–10^. Although principally confined to populations using more distal vs. proximal patterns of caregiving^11,12^, this early research showed the infant’s initial propensity for social engagement, with social interactions being characterised by periods of mutual gaze, and by parental responsiveness to infant social cues such as smiles, and oral and vocal communicative signals^1,3,6,13,14^. Although parental responsiveness occurs with similar frequency across cultures^15,16^, it varies in its form: in some cultures, caregivers typically respond by vocalising, smiling and showing exaggerated expressions (distal parental practices), while in others, caregivers tend to respond by rocking, caressing, kissing, patting, or repositioning their infants (proximal parental practices). Although the distal face-to-face interchanges are less frequent in societies providing more proximal parental care, they can still be observed in such cultural settings^10,11,17–19^; in fact, talking, smiling, showing exaggerated facial expressions to engage or respond to infants are considered part of the ‘intuitive parenting’ repertoire of behaviours^4^. Notably, while cross-cultural differences in distal vs. proximal caregiving practices have been reported at around three months of age^11,18^, observations of earlier mother-infant interactions suggest that the frequency of maternal responses to facial and vocal infant cues is not significantly different across-cultures before two months of age^17,20^.

Although relatively little research has been conducted on interactions during the period from birth to 2–3 months, studies have shown the development of infant social expressiveness to be influenced by particular parental behaviours^21–23^. In our previous work conducted on a sample of British mothers and their infants^23^, we showed that there is a ‘functional architecture’ to these early social exchanges—that is, mothers used specific responses to specific infant behaviours, and certain maternal responses ‘functioned’ to promote the development of infant social expressiveness. Specifically, by applying a micro-analytic coding scheme (i.e., second-by-second, including the coding of specific infant and maternal behaviours) and associated purpose-built software to identify the inter-dependencies between key infant expressive behaviours and maternal responses identified in the literature, and using prospective longitudinal observations of mother-infant face-to-face interactions recorded from birth through the first 2–3 months, we found that mothers showed significant specificity in their responsiveness, deploying particular behaviours (‘mirroring’, ‘marking’ and ‘negating’) in relation to different infant cues. Of particular note was the finding that maternal marking (where the mother shows emphatic, non-imitative responses to infant cues), and especially maternal mirroring (where the mother imitates infant behaviour, sometimes elaborating on it) increased infant social expressiveness, both concurrently and longitudinally^23^. A subsequent study with a similar British sample showed, moreover, that these mirroring responses influence later brain responsiveness to social expressions^24^. Such findings on the importance of maternal mirroring complement the wider evidence on the capacity of human infants themselves to imitate, or mirror, others’ facial gestures^25,26^, with the two lines of research converging to suggest common neural mechanisms underlying the capacity for experiencing self-other equivalence^27^. Notably, our subsequent cross-cultural and clinical research showed that this same fundamental *functional architecture* applied to mother-infant interactions in European samples with distinctive values concerning socio-emotional expressiveness (Italian and British), and even to dyads where infant facial expressiveness is affected by cleft-lip, and with the same effects on infant functioning^28–30^.

Given the importance of these parent-infant interactions for infant social development in our previous research^23,24,28–30^, a critical question, and one not previously investigated, is whether the same fundamental ‘functional architecture’ that we previously identified is found in groups of non-human primates too. Intuitive parenting behaviours, including providing supportive care, encouraging locomotion, playful interactions and contingent responses to infant social signals, have been described in many primate species, although differences in particular parenting styles depend on the rate of infant development, as well as the social structure^31^. For example, in chimpanzees, intuitive parenting is expressed in mutual interactions during which mothers engage in contingent behaviours and eye-to-eye contact with their infants^31–33^. These mutual gaze episodes seem to be inversely related to maternal cradling, suggesting that these interactions occur while mothers and infants are not in physical contact^32,34^, similar to what has been observed in human Western populations using a more distal parenting style^21^. As evidence of mutual gaze has been reported in other apes^35,36^, some have suggested that mother-infant face-to-face interactions probably emerged with the evolution of hominoidea (i.e., apes)^37^. However, while direct eye contact between adult monkeys often signals threat, several studies have reported mother-infant mutual gaze in monkeys too^38–42^. For example, mutual gaze episodes between adults and infant have been reported in some species of New World monkeys^43,44^. Notably, these visual exchanges can also include vocal elements: thus, in squirrel monkeys, from the first day of birth, infants engage in mutual gaze with adults and respond visually *and* vocally to vocalizations (i.e., caregiver calls) directed to them^43^.

More strikingly, in rhesus macaques, recent studies have revealed that mother-infant pairs exhibit socio-emotional interactions, including lip-smacking and sustained mutual gaze^39,45^. Experimental studies have shown that, like human infants, newborn rhesus macaques are attracted to faces, and in particular to the eye region^46,47^, and their social behaviours are influenced by mirroring, vs. other forms of contingent responses, performed by human adult social partners^48^. Notably, and as has been reported from research on Western human populations, it has been found that there is a link between this type of early social interactions and later development. Thus, infant macaques who have more frequent face-to-face interactions with their mothers subsequently engage in more social behaviours with their peers in their first year of life^41^.

Despite these important findings indicating areas of communality between rhesus macaque and human social development (as reported, predominantly for Western populations), we still know very little about the naturally occurring structure and development of mother-infant interactions in this monkey population: how do macaque mothers respond to their infants’ gestures? Do they actually mirror their infants’ behaviour and elaborate on their facial expressions and vocalizations, as has been described in populations of Western human mothers? Do they also mark their infants’ behaviour? We posit that a similar functional architecture to that described in humans is also present in non-human primates, in this case rhesus macaques, suggesting that social interactions between mothers and infants might have evolved much earlier than when apes first appeared.

To date, the comparative approach taken in most research on infant development has been subject to a number of limitations such as the lack (with few exceptions^39,41^) of naturalistic settings and of direct comparisons of the development of spontaneous mother-infant communication in human and non-human species. A novel comparative-developmental approach using naturalistic settings and comparable measures might be particularly valuable for highlighting any differences, as well as similarities, in parenting and developmental processes in primates, and for tracing the evolutionary roots of parenting behaviour.

The goal of the current study was to investigate, from an evolutionary and comparative perspective, whether the development and the organization of early infant communicative and affective behaviour, and the form of maternal responsiveness that we had identified in a population of British human mother-infant interactions also applied to a group of rhesus macaques (*Macaca mulatta*). In particular, we aimed to address three main research questions:

(i) Whether the form and structure of infant social expressiveness and of maternal responsiveness was the same in the rhesus macaques as it was in the human sample we studied—that is, whether the two groups showed comparable behaviours that entail the same patterns of relationship to each other;
(ii) Whether infant social expressiveness and maternal responsiveness in the rhesus macaque group followed a similar developmental pattern to that seen in the human group;
(iii) Whether the macaque mothers used the same kinds of response (mirroring and marking behaviours) as the human mothers in relation to the same specific set of infant behaviours.

In our previous work^23^, we had video-recorded human mother-infant face-to-face interactions at home from 1 to 9 weeks. In the current study, we used comparable behavioural data on face-to-face early interactions in rhesus macaques and compared them to the human data previously collected. Specifically, by using focal animal sampling in an outdoor setting, we video-recorded macaque mother-infant interactions occurring from the infant’s day of birth to 2 weeks. The ages chosen in monkeys are comparable to those in humans, given that development is approximately four time faster in macaques than in humans^49–54^ (i.e., a 2 day-old rhesus is comparable to a 8 day-old human, and a 2 week-old rhesus is comparable to a 2-month-old human), and that rhesus macaque mother-infant communicative exchanges are more frequent during the first two weeks of infant life, and significantly decrease after that time^39^. Further, during these two periods in both groups we have observed the emergence of the first affiliative behaviours and a steady increase of face-to-face exchanges^23,39^.

With regard to the coding of interactions, we used comparable coding schemes: thus, for the human infant sample, we had previously coded a comprehensive set of facial and vocal behaviours (proto-communicative mouth movements [i.e., mouth opening and tongue protrusion], smiles and vocalizations, as well as expressions of negative affect and non-social mouth movements), and in the current study we coded a comparable set of macaque infant facial gestures (proto-communicative mouth movements [i.e., mouth opening, tongue protrusion] and lip-smacking, together with vocalizations and non-social mouth movements). For maternal responses, in both human and macaque samples, and in line with previous research^23,55,56^, we recorded the presence/absence of a contingent maternal response to the coded infant behaviours within 2 s from the onset of infant behaviours, as well as its form, i.e., the two key maternal responses of mirroring and marking. By developing a common coding scheme and conducting a second-by-second analysis of the interactive sequences, we were able to compare mother-infant relationships in the two groups and describe their natural structure in a detailed and systematic way. In our previous work on human mother-infant interactions^23^, we had combined the different infant social behaviours (i.e., proto-communicative mouth movements and smiles), as well as the different kinds of maternal mirroring responses (i.e., ‘direct’, ‘enriched’ and ‘modified’). In the current study, we similarly used these same generic categories of infant and maternal behaviour. In addition, however, we adopted a more granular approach and retained the subcategories of each behaviour in our analyses. This was done because of possible differences in evolutionary development (including the developmental trajectories of communicative vs. affiliative infant behaviours) and previous descriptions of different forms of mirroring response^16,57–59^ indicating that they may serve different functions in mother-infant communication. Further, this more granular approach allowed us to better address our third aim of examining the extent of specificity in the way interactions were structured in the two groups (see Methods section for more details).

## Results

### The structure of infant and maternal behaviours—PCA

In the human sample, one component was extracted that explained 42.97% of the variance in infant behaviour (*KMO* = 0.693; Bartlett's Test of Sphericity *Χ*^*2*^ (10) = 64.398, *p* < 0.001): behaviours with absolute value loadings > 0.5 were Proto-communicative Mouth Gestures, Vocalisations, and Smiles, i.e., positive social behaviour. Non-social mouth movements, and expressions of negative affect were found to load negatively on the component, albeit below the 0.5 threshold (Table 1).

**Table 1:** PCA on Human behaviour.

Infant behaviours	
Proto-communicative mouth gestures	0.809
Positive vocalisations	0.775
Smiles	0.716
Non-social mouth movements	− 0.481
Negative affect	− 0.387
Maternal responses	
Marking	0.831
Mirroring	0.797
Negative responses	0.320

With regard to maternal responses, one component explained 47.62% of the variance (*KMO* = 0.506; Bartlett's Test of Sphericity *Χ*^*2*^ (3) = 14.970, p = 0.002). Considering absolute value loadings > 0.5, the component included Mirroring and Marking. Negative responses loaded only weakly on the identified component (Table 1).

In the infant macaque sample, one component was extracted that explained 42.18% of the variance (*KMO* = 0.459; Bartlett's Test of Sphericity *Χ*^*2*^ (6) = 20.598, *p* = 0.002): behaviours with absolute value loadings > 0.5 were Proto-communicative Mouth Gestures, and Lip-smacking, i.e., positive social behaviour. Non-social Mouth Movements, and Negative Vocalisations loaded only weakly on the identified component (Table 2).

**Table 2:** PCA on Rhesus macaque behaviour.

Infant behaviours	
Proto-communicative mouth gestures	0.920
Lip-smacking	0.904
Negative vocalisations	0.120
Non-social mouth movements	0.101
Maternal responses	
Mirroring	0.880
Marking	0.880

For macaque maternal behaviour, one component was extracted that explained 77.45% of the variance (*KMO* = 0.500; Bartlett's Test of Sphericity *Χ*^*2*^ (1) = 10.580, *p* = 0.001), and showed both Mirroring and Marking with loadings > 0.5 (Table 2).

Given the theoretical distinction between mirroring and marking^23^, we retained both these maternal responses comprising the first component as separate variables for data analyses, despite their empirical association.

### Developmental trajectory of interactive behaviours in humans and monkeys

The mean time of mother-infant interactions coded for each group, for the different time periods observed was M = 146.05 s (SD = 47.03) in humans, and M = 30.91 s (SD = 31.79) in monkeys.

#### Mutual gaze

**Figure 1:**
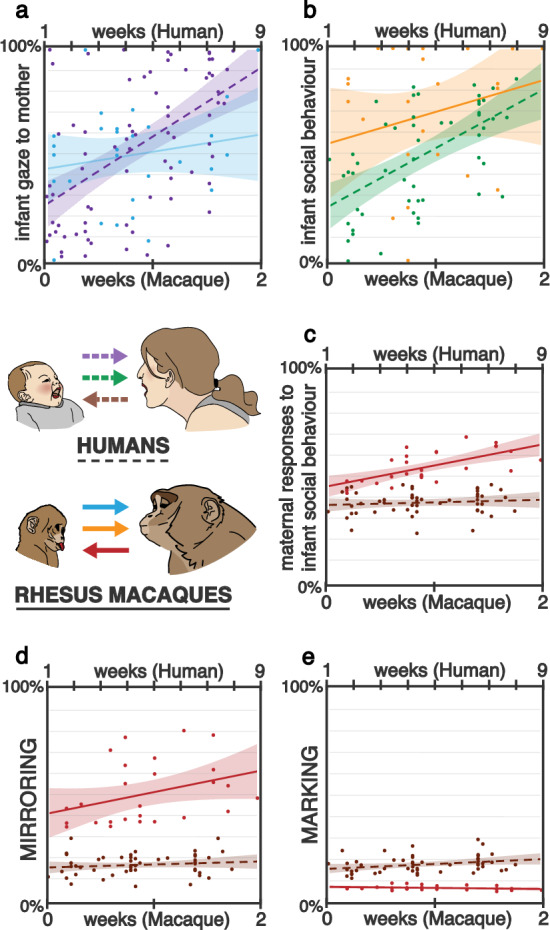
Comparative change in infant and mother behaviours in human (dotted lines) and rhesus macaque (solid lines), according to infant age (human at the top, rhesus macaque at the bottom; ratio of development of rhesus macaques to humans is 4:1). (**a**) Change in infant gaze to mother (as percentage of interaction time) in humans (purple) and macaques (blue); (**b**) Prevalence of infant social behaviours (as percentage of all behaviours) in humans (**a** green) and macaques (yellow); (**c**–**e**) Maternal responsiveness to infant social behaviours (**c**) as estimated percentage of all social infant behaviours) in humans (brown) and macaques (red): maternal mirroring response (**d**) and maternal marking responses (**e**; as estimated percentage of maternal responsiveness to infant social behaviours). *Note* In all panels, shaded areas represent 95% confidence intervals and each datapoint represents a single observation. As described in the text, results shown in (**c**–**e**) focused on human infants aged 5 weeks and older, with the removal of 35 data points, compared to (**a**) and (**b**), which instead also included weeks 1 and 3.

The time infants spent looking at their mother (as a proportion of the total time of interaction) was compared between human and rhesus macaques (Fig. 1a). A significant interaction between age and group emerged (*Χ*^*2*^ (1) = 49.744, *p* < 0.001), with only human infants showing a significant increase in time looking towards the mother over the period assessed (*p* < 0.001), while no such increase was found in rhesus macaques (p = 0.468).

#### Social expressiveness

Human and rhesus macaque infants were compared according to the change in their social behaviours, represented as a percentage of the total numbers of behaviours displayed (Fig. 1b). To enable comparisons, age in days was standardised: for each group, their mean value and standard deviation for age were used to compute a new variable having mean value set at 0 and standard deviation equal to 1. For human infants, only data from the 5-week visit onwards were considered, (with the removal of 35 data points, corresponding to weeks 1 and 3), as this was when social behaviours first appeared in this group.

A significant effect of group emerged (*Χ*^*2*^ (1) = 7.318, *p* = 0.007), with rhesus macaque infants generally showing higher percentages of social vs non-social behaviours than human infants. A significant main effect of age also emerged (*Χ*^*2*^ (1) = 7.948, *p* = 0.005), with the percentage of social behaviours generally increasing over time in each group. Finally, a significant interaction between age and group emerged (*Χ*^*2*^ (1) = 5.157, *p* = 0.023). As shown in Fig. 1b, the increase in social behaviour was significant in the human group (p < 0.001), but not in the rhesus macaques (p = 0.750). Moreover, while the group difference was significant (p = 0.001) at the youngest infant ages considered, it was no longer so when infants reached the oldest age analysed (p = 0.734).

#### Maternal responsiveness

The percentages of infant behaviours (both social and non-social) that were responded to by the mother, in each group, were compared, controlling for the general rate per minute of infant behaviours, to account for the level of stimulation mothers received. A main effect of age emerged (*Χ*^2^ (1) = 5.345, p = 0.021), showing that, regardless of group, maternal responsiveness increased over the period analysed. A main effect of group also emerged (*Χ*^2^ (1) = 12.098, p < 0.001), with human mothers showing generally lower levels of responsiveness, compared to rhesus macaques. The interaction between group and age was not significant.

To investigate maternal responsiveness to infant *social* behaviour specifically (Fig. 1c), we repeated the above model, including only this kind of infant behaviour in the analysis. The effect of the ratio between social behaviours and all infant behaviours was controlled for, to account for the prevalence of social behaviours within each infant’s corpus of behaviours. Age in days was standardised within each group (as specified above for social expressiveness) and, for human infants, only data from the 5-week visit onwards were considered. Only a significant effect of group emerged, with rhesus macaque mothers responding to infant social behaviours to a greater extent than human mothers (*Χ*^2^ (1) = 5.681, p = 0.017).

As well as the extent of maternal responsiveness to infant social expressions, we investigated its form by comparing the occurrence of the different categories of maternal response (i.e., mirroring and marking) in the two groups. To do so, models similar to the one above were conducted, including only the target kind of maternal response, and showed the results below.

#### Maternal mirroring responses

Human and rhesus macaque mothers were compared in relation to their use of mirroring responses to their infant’s social behaviours. Mirroring was thus represented as the percentage of infant social behaviours that received this response, out of all social behaviours displayed by the specific infant. To account for the effect on maternal mirroring of the prevalence of social behaviours within each infant’s corpus of behaviours, the model examining mirroring controlled for the effect of the ratio between social behaviours and all infant behaviours.

A significant effect of group emerged (*Χ*^*2*^ (1) = 22.773, *p* < 0.001), with rhesus macaque mothers generally showing higher percentages of infant social behaviours being mirrored than human mothers (Fig. 1d). A main effect of age failed to reach significance (*Χ*^*2*^ (1) = 2.764, *p* = 0.096), with the percentage of social behaviours being mirrored seemingly increasing over the period of time considered for each group. The interaction between age and group was not significant (*Χ*^*2*^ (1) = 0.091, *p* = 0.763).

#### Maternal marking responses

Finally, when analysing maternal marking responses, no main effect of age was found, but a significant group effect emerged (*Χ*^*2*^ (1) = 7.318, *p* = 0.007), with higher proportions of infant social behaviours eliciting this kind of maternal response in the human mothers, compared to the rhesus macaque mothers, who showed very low levels of marking response (Fig. 1e). No interaction between age and group was found.

### Specificity of maternal mirroring responses to different infant behaviours

**Figure 2:**
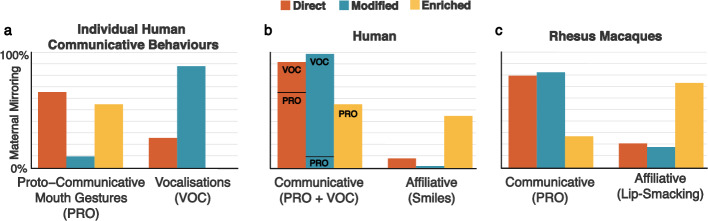
Type of Maternal Mirroring response for each infant social behaviour. Direct (orange), Modified (blue) and Enriched (yellow) mirroring in response to infant communicative behaviour (proto-communicative mouth gestures and vocalizations) in humans (**a**) and social behaviour (communicative and affiliative) in humans (**b**) and in rhesus macaque (**c**). *Note* Within each panel, and for each kind of mirroring responses, bars represent the percentage of the total corpus of that kind of mirroring responses that was used in response to the specified kind of infant behaviour (e.g., in (**b**), which includes all infant behaviour categories, orange bars add up to 100% of direct mirroring responses).

The previous analysis examined the broad categories of maternal response, namely mirroring and marking. In the next analysis, we investigated whether infant behaviours differentially elicited the separate subcategories of mirroring response (Direct, Enriched and Modified). Thus, for each infant behaviour that elicited any maternal mirroring, we compared the percentages of occurrence of each type of maternal mirroring response, controlling infant age and the base rate of the given infant behaviour (Fig. 2). This approach allowed us to test whether each kind of maternal mirroring response was used proportionally differently in response to the various categories of infant behaviours (e.g., whether the percentage of direct mirroring responses that was used for infant proto-communicative mouth gestures, out of all direct mirroring responses, differed from the percentage of modified mirroring responses that was used for the same infant behaviour, out of all modified mirroring responses).

In humans, infant Proto-communicative mouth gestures elicited different percentages of the different kinds of mirroring (*Χ*^*2*^ (2) = 49.718, p < 0.001), with lower percentages of mothers’ Modified Mirroring responses (10.42%) being used, compared to both their Direct (65.52%) (p < 0.001), and Enriched Mirroring (54.90%) (p < 0.001) (Fig. 2a). For infant Vocalisations, Enriched Mirroring was not included, as this maternal response was not shown. Mothers’ use of Direct and Modified Mirroring differed from each other in response to vocalisations, with a higher percentage of occurrences of the latter (87.50%) being used than of the former (25.86%) (*Χ*^*2*^ (1) = 31.436, p < 0.001) (Fig. 2a). Given the shared communicative function of proto-communicative mouth gestures and vocalisation, we pooled these two infant behaviours in a single Communicative behaviour category, and repeated the previous analyses. Overall infant Communicative behaviour was, once again, found to elicit different percentages of the different kinds of mirroring responses (*Χ*^*2*^ (1) = 37.944, p < 0.001), with a smaller percent of occurrences of Enriched Mirroring (54.90%) being used than of both Direct (91.40%) (p < 0.001) and Modified mirroring (97.90%) (p < 0.001), with no difference between the latter two (Fig. 2b).

Differences between types of maternal mirroring also emerged in relation to Smiles (*Χ*^*2*^ (2) = 28.349, p < 0.001), with a higher percentage of mothers’ Enriched Mirroring responses (45.10%) being shown to this affiliative infant social behaviour, compared to both Direct (8.62%) (p = 0.054), and Modified Mirroring (2.08%) (p = 0.024) (Fig. 2b).

In rhesus macaques, mirroring was used only in relation to proto-communicative mouth gestures and lip-smacking; for each of these two infant behaviours, differing percentages of each of the three kinds of maternal mirroring response were produced (*Χ*^*2*^ (2) = 8.160, p = 0.017). For proto-communicative gestures, a higher percentage of mothers’ Modified Mirroring (82.14%) was used compared to their Enriched Mirroring responses (27.27%) (p = 0.035), with also a higher percentage of Direct Mirroring responses (79.17%) compared to Enriched mirroring failing to reach significance (p = 0.072). By corollary, the same results, albeit in the opposite direction, applied to the rhesus macaque affiliative behaviour of lip smacking (Fig. 2c): a lower percentage of mothers’ Modified Mirroring (82.14%) was used compared to their Enriched Mirroring responses (27.27%) (p = 0.035), with also a lower percentage of Direct Mirroring responses (79.17%) compared to Enriched mirroring failing to reach significance (p = 0.072).

As can be seen comparing panels b and c in Fig. 2, the pattern of distribution of the different kinds of maternal mirroring appeared strikingly similar for humans and monkeys for both communicative (proto-communicative mouth gestures plus vocalisations for humans, proto-communicative mouth gestures for rhesus macaques) and affiliative (smiles for humans, lip-smacking for rhesus macaques) behaviours. In a final analysis, similar in model design to the ones reported above, but with the inclusion of the effect of group, we therefore examined the occurrence of maternal mirroring responses to infant communicative and affiliative behaviours across the two groups, still controlling for the effects of infant age and infant behaviour base rate. Across groups, infant communicative behaviours elicited higher percentages of Direct and Modified Mirroring responses than of mirroring responses of the Enriched kind (both p < 0.001), and seemingly higher percentage of Modified mirroring than of Direct Mirroring responses although this last difference failed to reach significance (p = 0.082) (*Χ*^*2*^ (2) = 36.433, p < 0.001). Nevertheless, a significant main effect of group also emerged (*Χ*^*2*^ (1) = 7.322, p = 0.007), with the rhesus macaque mothers showing higher percentages of the three kinds of mirroring responses to affiliative infant behaviours, compared to the human mothers, who, complementarily, showed higher percentages to infant communicative behaviours. Notably, the interaction between kind of mirroring and group was not significant.

## Discussion

This study provides a detailed, systematic description of the structure of naturally occurring early mother-infant interactions in rhesus macaques and aims to directly compare the development of infant social expressiveness and maternal responsiveness between a group of these non–human primates and a group of human mother-infant pairs from a British population, using a common micro-analytical coding scheme. Our findings show, on the one hand, that the mother-infant relationship was characterized by a number of common features across the two primate groups and, on the other hand, that the development of the early communicative system between mothers and infants showed specific variations across these same populations.

Our PCA analysis showed a consistent structure of infant social behaviours and maternal responses across the two groups. In both, we identified one component for infant behaviours. As described by Murray et al.^23^, human infant social expressiveness is highly structured, combining different individual behaviours (i.e. proto-communicative mouth movements, smiles, and positive vocalizations) in a single group (i.e. social behaviours), distinct from non–communicative mouth movements and expressions of negative affect. This was similarly the case in our macaque sample, where the single component identified included strong loadings for proto-communicative mouth gestures and lip-smacking, but not for non-social mouth movements and negative vocalisations. With regard to maternal behaviour, moreover, in both groups, maternal responses, i.e., mirroring and marking behaviours, loaded on the same component. To our knowledge, no previous description of these behaviours being deployed by macaque mothers during naturally occurring face-to-face interactions has been reported. Together, these results show a common structure to early social interactions across our samples of human and macaque mother-infant pairs, suggesting that mirroring and marking responses might represent a common type of parental responsiveness, occurring not only in humans but also in other primate populations.

In spite of striking commonalities, and through methods that took into account the different developmental rates between the two species, we identified important differences in the developmental timing of infant social expressiveness and of maternal responsiveness between the two groups. Thus, while our human sample results showed an overall increase over time in the expression of infant social behaviours and in maternal responsiveness, in line with previous studies on Western samples^9,23^, no such changes across time were identified in the rhesus macaque group. This difference reflected the fact that, whereas in the rhesus macaque infants both the percentage of time spent in visual engagement and the percentage of social vs. non-social behaviours was remarkably high from the first week, in the human group of infants, visual attention to mothers was very low in the first three weeks and rapidly increased thereafter (Fig. 1a). This result is in line with previous studies showing that in Western middle-class populations, the percentage of time spent in mutual gaze between mother and infants increases across the first three months^17,21^ as a result of maternal encouragement of face-to-face episodes during distal interactions^21,32^. Similarly, the appearance of infant social signals in the human group in our study started only from five weeks (Fig. 1b), albeit rapidly increasing over time and reaching, at around 2 months, a rate similar to the one found in the rhesus macaques at 2 weeks. This result is consistent with previous cross-cultural studies conducted in the first three months. These show that in cultures where infant social smiles and vocalisations are responded to, and therefore promoted, the expression of these behaviours increases in rate and duration from 2 to 3 months of age; conversely, in cultural contexts where proximal forms of communication are more prevalent (e.g., touch), face-to-face contact is reduced and responding to these infant social behaviours is not prioritised there is, correspondingly, no change in their duration and frequency across age^17,22,60,61^. It is worth noting that although researchers have characterised such findings purely in terms of ‘cultural’ differences, other contextual factors (i.e., maternal level of education) might also contribute to such differences in socialisation practices^17,62,63^. Accordingly, further studies on early social interactions should investigate how contextual factors such as maternal education and socio-economic status can affect socialisation goals both within and across cultures.

The difference in the emergence and development of infant social expressiveness between the human and the monkey groups in our study is consistent with comparative and evolutionary studies showing that human infants are born early relative to their stage of neurodevelopment compared to other primates^64–66^. In fact, while apes and monkeys experience very rapid brain growth in utero with a slowdown around the time of birth, in humans brain growth continues at foetal rates for most of the first year of life^67,68^. Portmann^69^ coined the term “secondary altriciality” to describe the distinct state of human neonates compared with the primary or primitive altriciality of mammals with precocious development. The shortening of human gestation and the extensive neural and cognitive maturation that takes place in the first year of life could explain why the expression of salient infant social signals, such us sustained social gaze, smiling, and non-distress vocalizations, emerge well after the first month. This longer maturation of human infant social expressiveness is held to have important implications for parental behaviours and social relationships. In fact, one proposed function of extended immaturity and associated plasticity, is that it allows more time and opportunities for learning and the flexibility necessary to master skills required for living in highly complex and variable societies^70–74^. In this regard, it has been suggested that imitation of facial expressions between mothers and infants may be evolutionarily adaptive as it allows children to acquire the particular cultural information that is important for their social group^75^. Our results in the current study, together with those for a contrasting Italian sample, support this argument, as each group of mothers selectively mirrored and reinforced those particular infant behaviours that align with British and Italian cultural expectations and goals^28^. Such ‘intentional’ selectivity where mothers direct their responses toward specific behaviours, rather than evenly distribute them across all infant actions, thereby provides infants with targeted opportunities for acquiring culturally important skills and behaviours. Similarly, in our sample of rhesus macaques, maternal mirroring was used in response to important social signals (i.e., lip-smacking), which in this species are crucial to effectively communicate positive intentions and affiliation to others^76–80^. Due to their faster brain growth and rapid development of cognitive and socio-emotional behaviours compared to humans^52^, along with the crucial role of facial communication in despotic species^81^, where tolerance is low and social hierarchy is highly structured, the learning of these affiliative gestures might have been prioritized in evolution, thus supporting the high frequency and specificity of these exchange signals in mother-infant communication. In fact, in the first month of macaque life, individuals are already capable of independently moving in their environment and engaging socially with other adults or peers^78,80,82,83^, and therefore the ability to appropriately respond to, as well as effectively perform social signals, is crucial for their social relationships. The importance for macaque social development of being exposed to these interactions from very early on in life is further supported by studies showing how the lack of these early exchanges between mothers and infants can lead to detrimental outcomes^84–90^.

In line with previous research^91–95^, our results showed that, differently from the infant macaques, even in the first weeks of life, our sample of human infants produced positive and neutral vocalisations during face-to-face interactions with their mothers. Such sounds, although devoid of any language structure, have been argued to comprise the foundations for all subsequent vocal development necessary for language, including canonical babbling^92,96^. Interestingly, these neutral/positive vocalizations have recently been described in infant bonobos^97^, suggesting an evolutionary foundation of human language in non-human primates. However, unlike in human mothers, where responsiveness to these infant signals occurs, in bonobos, maternal vocalizations are not directed toward the infant. Although in previous studies, a positive vocalization (i.e., girn) has been described in 4–7 month-old infants macaques in response to reunion with the mother after separation^98^, in our study, no such vocalizations were identified in the macaque infants – possibly due to the younger age of our subjects, or because all face-to-face interactions between mothers and infants occurred in close proximity^39^ – and therefore no maternal response to them was required. That said, it is worth noting that both bonobo mothers and macaque mothers are not unresponsive to infant vocalizations. Indeed, they respond quickly and comfortingly to them, for example by looking toward the infant, picking and holding them up, or through facial expressions^97,98^. Interestingly, vocal exchanges between mothers and infant have been reported in some species of New World monkeys (i.e., squirrel monkeys, common marmosets)^43,44^. For example, in squirrel monkeys, it has been reported that from the first day of birth, infants respond visually and vocally to adult (mostly allomothers’) vocalizations directed to them^43^. These vocal exchanges mostly occur when infants and adults are engaged in mutual gaze, and modulation of their frequency is greatest during eye contact, resembling the melodic intonation contours used during vocal responses by human mothers to prelinguistic infants. Most interestingly, these vocal exchanges occur five times more often between infants and ‘aunts’ (other mothers within the social group) than between mothers and infants. The reason for this special role of allomothers is related to infant position, carried on the mothers’ back, thus preventing mothers from engaging in eye contact with their infants. This specific habit of vocalizing to an unrelated infant on someone else’s back may best be understood as a reciprocal solution to the difficulty of making eye contact with one’s own infant. As for humans, for squirrel monkeys the most obvious benefit of eye contact and associated behaviours is to facilitate vocal interactions, which is the most prominent form of communication in this species^99^. Taken together, this evidence might suggest that, although maternal responsiveness to infant social behaviours appears to be a universal feature of mother-infant interactions, maternal *vocal* responsiveness might be an evolutionary adaptation linked to the specific social structure and parental practice used by different primate species, with cooperative caregiving and alloparenting likely to play an important role. Data on maternal vocal responses to infant positive vocalizations in non-human primates are scarce, so future studies should investigate these behaviours in different primate species and examine the role of different social structures and caregiving practices across species in eliciting maternal vocal responses.

Another important aspect of vocal interactions between mothers and infants identified in the current study was the specific maternal response used by the human mothers toward positive vocalizations. Thus, using a granular categorisation of maternal mirroring, our results revealed that infant positive vocalizations reliably elicited *modified mirroring* responses (Fig. 2a,b), whereby mothers perform their own version of the infant’s behaviour, typically changing it into a more prototypical or socially meaningful form (Fig. 4). This not only replicates previous findings that infant non-distress vocalizations tend to elicit vocal/verbal responses from mothers^100–102^, but also suggests a specific form of maternal response. Recent evidence indicates that infant vocal learning is embedded in a social feedback loop^103,104^, and that infants use social feedback to facilitate developmental transitions in vocal behaviour^105–107^. Interestingly, an increasing body of evidence suggests that, despite variations in the temporal coordination and timing of vocalizations across different cultures^15,17,108,109^, as well as in the frequency of vocal interactions^110–114^, conversational exchanges between mothers and their young children nevertheless do occur across populations with different languages (tonal vs non-tonal) and different cultures (Western vs non-Western), and are characterized by similar features in terms of pitch contours, rhythm, intensity and repetitiveness^9,57,115–118^. Moreover, vocal imitation of infant vocalizations and its role in stimulating and reinforcing specific vocal expressions^57,105,119,120^, therefore facilitating vocal learning^105,120,121^, has been reported in an increasing number of human populations^122–129^. Our findings can be interpreted within such a framework, suggesting that, in populations (both human and non-human) where vocal exchanges between mothers and infants occur, modified mirroring may constitute a form of pre-linguistic communication in which caregivers provide structured feedback to their infant’s early vocalizations, therefore creating new opportunities for vocal learning. In line with previous evidence on the role of maternal vocal responses to infant vocalizations in both Western and non-Western populations^106,107^, our findings suggest that a process of co-regulated interaction is at work very early in development, and that modified mirroring responses to infant early vocalizations might gradually shape infants’ patterns of communication in *culturally* specific ways, as well as reinforce and motivate their vocalizations. In order for our results to be generalized to other populations, more cross-cultural studies need to be conducted to investigate the extent to which these maternal behaviours are shared across different cultures, and if so, whether they share a similar form and function in relation to language development. Such studies combined with comparative ones in non-human primates would enable a deeper evaluation of the role of vocal maternal responses in the emergence and development of language in human infancy.

From a comparative point of view, it is worth noting that modified mirroring, used by our sample of human mothers to respond to infant vocalizations, was also used by the macaque mothers in response to infant proto-communicative facial gestures (alongside the simpler category of direct mirroring) (Fig. 2c). In particular, the macaque mothers were observed to respond to infant mouth openings by lip-smacking, a more socially meaningful communicative gesture (Fig. 4), and by encouraging the repetition of this gesture in the form of a low-frequency lip-smacking. Aside from its affiliative function, the production of lip-smacking in macaque monkeys is strikingly similar in terms of its form—likely homologous—to the orofacial rhythms produced during speech^130^, as well as in terms of its developmental trajectory^131^ and the coordination of vocal tract structures^132,133^, and it seems to activate lateral frontal areas homologous to Broca’s area^134^. Our results are therefore in accordance with the evolutionary theory that posits that during the course of speech evolution, such non-vocal rhythmic facial expressions were coupled to vocalizations to produce the audio-visual components of babbling-like (i.e., consonant–vowel–like) speech expressions^135,136^ in the service of early mother-infant vocal communication.

A further remarkable similarity between the human and rhesus macaque groups revealed by our findings was the use of *enriched mirroring* responses by mothers following infant smiles and lip-smacking, respectively (Fig. 2). Both these infant communicative gestures share a similar reward value in the two species^137–140^ as they both involve an emotional component and seem to advertise cooperative dispositions and affiliation^20,141,142^, thereby increasing the likelihood of engagement in social interactions.

In both Western and non-Western human populations, the emergence of social smiles coincides with the emergence of sustained mutual gaze between mothers and infants around 6 weeks, although sociocultural factors seem to affect its development during the 2-month shift. In fact, while in Western populations the development of social smiling after 6 weeks is affected by maternal affective mirroring during mutual interactions, in non-Western populations an increase in infant social smiles mediated by maternal imitation of this behaviour occurs *after* 3 months, in line with their different cultural expectation of the emergence of joy in infants^60^. In populations of Western middle-class mothers, maternal affective mirroring has been linked not only with the subsequent emergence of infant smiling but also with sequences of positive feedback between infant and maternal emotional expressions. In line with the socialization goals reported in these populations, by using their own emotional expressions to respond to the one shown by the infant, mothers seem to encourage their infant’s emotions, thus providing them with continuous feedback about what kinds of emotion and emotional expression are appropriate in different contexts^9,14,28,102,143^. As noted, in both groups we studied, maternal mirroring responses to these gestures were often of the ‘enriched’ form, that is, they were accompanied by some elaboration in a different modality (Fig. 2a–c). For example, our sample of human mothers, in addition to simply matching their infant’s smile with direct mirroring, responded to them not only by imitating their smile but also adding some vocal response or exaggerated facial expression (Fig. 5a,b). Similarly, the macaque mothers, when responding to infant lip-smacking, often imitated the gesture and also accompanied it with exaggerated body postures, head bobbing movements or teeth chattering and silent bared teeth (Fig. 5c-e). By deploying an affiliative gesture coupled with an additional signal of prosocial intention, macaque mothers effectively advertise their willingness to engage in a mutual interaction, as well as shape the infant’s ability to identify individuals who are prepared to do so later in development. Taken together, our observations are in agreement with the intersensory redundancy hypothesis postulated by Bahrick and Lickliter^144^, claiming that both animal and human infants are especially proficient at detecting multimodal, redundant stimulation, and detection of this information can organize early attention and provide a foundation, and guidance for perceptual development^144–146^. Indeed, within the context of face-to-face interactions, adults regularly scaffold infants' attention and provide a rich interplay of concurrent visual, vocal, and tactile stimulation. On this basis, the enriched mirroring responses used by mothers in both groups might be crucial in attracting the infants’ attention to a very salient communicative and affiliative gesture and reinforcing its expression. Despite the fact that mothers from different populations respond differently to different infant social signals, in line with cultural differences in parental practices^128,147–149^, infant-directed communication across different populations is multisensory and involves a wide range of auditory, visual and tactile information^150,151^. As reported in several cross-cultural studies, the integration of different modalities during early interactions facilitates infant attention^152–154^ and promotes learning^155–158^, thus suggesting the existence of a common process that nonetheless allows for the achievement of culturally-specific socialization goals.

Finally, we observed that along with mirroring behaviours, both the human and monkey mothers deployed another form of maternal response, i.e., marking behaviours, during engagements with their infants. Although mirroring and marking both loaded on the same component, the literature on these responses identifies important distinctions. Thus, while mirroring of the infant’s behaviour has been seen as potentially strengthening, or forging neuronal circuits tuned for decoding social information, marking has been highlighted principally for its functional, ostensive, role in assisting infants of 6-months and older to effectively respond to referential communication directed to them^159^. Although the human mothers used mirroring and marking at similar (and relatively infrequent) rates in response to infant social behaviours, it was notable that macaque mothers showed a considerably lower rate of marking relative to mirroring behaviours. In our previous study^23^, we suggested that marking behaviours deployed during early face-to-face interactions could represent a precursor of the later occurring ostensive behaviours crucial for the establishment of shared reference in triadic interaction and joint attention. Thus, in its earlier instantiation, maternal marking identifies the infant behaviour in question as the event whose significance is to be noted and shared by both partners. Accordingly, the remarkable difference in this type of maternal response between our samples of humans and monkeys might suggest that, while sharing important features of maternal responsiveness, the two groups significantly differ in the expression of specific maternal responses linked to the development of secondary, or referential, intersubjectivity – a unique trait of ape and human development^160^. This assumption is supported by evidence of behavioural marking among chimpanzees: during play, infant smiles are sometimes marked by the mother with an emphasized touch, and when the infant smiles in response to a tickle, the mother may place her index finger on the infant's lower gums and exaggerate the smile by pushing gently on the lower gums^161^. Interestingly, and similar to human populations beyond three months^162^, marking responses in Apes are conveyed through tactile and body stimulation, thus suggesting that the development of triadic interactions might not be solely mediated by visual engagement, but that other modalities, forms of attention and coordination might be used as precursors of joint engagement^12^. In our study, marking responses were coded only as conveyed through facial expressions, therefore an alternative explanation for the lack of evidence of marking behaviours in our sample of macaques might be that this type of maternal response *is* present in rhesus macaques too, but is mediated through a different sensory modality (i.e., tactile responses), similar to what has been reported in chimpanzees. Future studies should therefore further explore different forms of marking behaviours in human populations as well as in different primate species and identify cultural/species-specific variations in this type of responses and how these are related to the development of joint engagement.

In conclusion, our developmental-comparative approach to studying mother-infant early communication in humans and monkeys provides new important insights on the developmental trajectory of infant social expressiveness and maternal responsiveness in these two groups, and contributes to a better understanding of the evolutionary roots of parenting behaviours. By using data from naturalistic observations and a common, detailed coding scheme, we were able to identify a shared *functional architecture* of mother-infant interactions between humans and monkeys as well as those characteristics that are unique to the human group. Specifically, we showed that the differences between the human and the macaque group were of a quantitative rather than qualitative nature, as evidenced by the similar structure and pattern of maternal responses to infant behaviours.

More work is needed to fully understand differences and similarities in the development of early social interactions across species. Our study compared a group of British mothers a group of rhesus macaques, so results might not be generalisable to other human populations and primate species. As already discussed, socio-cultural factors can influence the type of interaction between mothers and infants, therefore future studies should include mothers from different cultural contexts to better capture shared features of early communication, as well as intraspecific variation, thereby improving the generalisability of our results. Similarly, among non-human primates, maternal behaviours differ widely across species, and different social structures and caregiving practices might influence the way mothers interact with their infants. Therefore, in order to further trace the evolutionary roots of parenting behaviours, future studies should include observations from different species living in different social contexts. Moreover, as the current study is the first systematic description of mother-infant interactions in rhesus macaques, further data on this species are needed in order to confirm our results. It should be noted that our data collection methods differed somewhat between the human and monkey samples, with the human data collected in a setting where mothers were asked to interact with their infants potentially explaining the longer duration of the face-to-face interactions in the human group compared to the macaque one. Therefore, in future studies, spontaneous human mother-infant face-to-face interactions during home observations, similar to those coded in macaques in this study, should be collected in order to provide more consistent data on the development and structure of these interactions and increase cross-species comparability. Finally, longitudinal observations combining a developmental, cross-cultural and cross-species perspective should be conducted in order to investigate the role of specific maternal responses in later infant development. In particular, future studies exploring the role of maternal responsiveness to infant vocal signals in different primate species could provide important information on the evolution of language and social communication.

## Methods

### Participants

#### Human subjects

Mothers of healthy full-term infants were recruited on the postnatal ward of the Royal Berkshire Hospital, Reading, UK, to a pool of volunteers for child development research at the University of Reading. Twenty mother-infant dyads (12 male infants) participated in the study. Infant ages in weeks at the five study assessments were M(SD) 1.47(0.29), 3.09(0.31)), 5.23(0.41), 7.01(0.33)) and 9.14(0.43), and the number of completed, codable assessments per infant was M = 4.15 (SD = 0.75). Reading is representative of the British population, and our sample was accordingly broadly in line with British demographics (albeit excluding conditions associated with high socio-economic risk (e.g., premature delivery) as well as major infant congenital disorders): maternal ages ranged from 28.74 to 41.11 years (M = 33.70 (SD = 2.73), one mother was single, (5%—vs. 12.5% British rate for single mothers); 60% were university graduates (compared to 52% British tertiary education average), two thirds (65%) were multiparous, and 90% were White (compared to 82% British average). Infant gestation was M = 40.79 weeks (SD = 1.59), and birthweight was M = 3731.94 gm. (SD = 608.07). All methods were carried out in accordance with the British Psychological Society’s Code of Human Research Ethics. All study protocols were approved by the Ethics Committee of the University of Reading (n. 11/45). Informed consent was obtained from the all the mothers to include themselves and their infants in the study.

#### Rhesus macaques

Rhesus monkey mother–infant dyads (*N* = 9; 3 male, 6 female infants) were born and raised at the Laboratory of Comparative Ethology’s NIH Animal Center in Poolesville, Maryland. All infants were reared by their biological mothers (aged between 5 and 12 years; 2 mothers were primiparous). All infants had been carried to term and born without further complications; birth weight for all infants fell within normal parameters (M = 520.00 gr, SD = 83.10). Infant ages in days in weeks 1 and 2 were M(SD) 4.17(2.08) and 11.12(1.64). Animals were housed in social groups containing each eight to ten adult females (including the infant’s mother), one or two adult males, and other similarly aged infants. Monkeys were housed in indoor-outdoor enclosures measuring 2.44 × 3.05 × 2.21 m indoor and 2.44 × 3.0 × 2.44 m outdoor. We studied dyads in the birth seasons (spring and summer) of 2011 and 2012. All testing was conducted in accordance with regulations governing the care and use of laboratory animals, and had prior approval from the Institutional Animal Care and Use Committee of the Eunice Kennedy Shriver National Institute of Child Health and Human Development.

### Procedure

#### Human subjects

In home visits at 1, 3, 5, 7 and 9 weeks postpartum, 3 min of mother-infant face-to-face interaction were video-recorded. Infants were placed semi-reclined on a mat on the floor, and mothers sat opposite, leaning towards their infant. When the infants were calm and alert, and ready to engage, mothers were asked to interact with their infants as they would normally do. A camera filmed the infant’s face and upper body, and a reflection of the mother’s face and upper body in a mirror placed behind the infant; another mirror placed alongside the infant showed their face if they turned away from the camera. A total of 83 episodes of face-to-face interactions was observed and coded.

#### Rhesus macaques

Behavioural data were collected on mother-infant dyads housed in indoor-outdoor enclosures in social groups (one adult male, several females and offspring). All observations were made when animals were in the outdoor portion of their indoor-outdoor enclosures. Using focal animal sampling, we recorded mother-infant interactions occurring from the infant’s day of birth to 2 weeks. Each pair was filmed between 09:00 and 17:00 h, one to two times per day, 2 to 3 days per week for 15-min sessions (for more details see Supplementary Table S2). In each 15-min session, we recorded all mother-infant interactions including face-to-face interactions. When a face-to-face interaction occurred, we identified the onset and offset of the interaction and coded it as described in the *Coding* section below. We discarded episodes of face-to-face interactions if either the infant’s or the mother’s face was not visible, as well as nursing episodes. A total of 66 episodes of face-to-face interactions was observed and coded.

### Coding

For coding purposes, face-to-face interactions between mothers and infants in rhesus macaques were defined as starting from the second when the infant made eye contact with mother and ending when both the infant and the mother stopped engaging with each other for more than 5 s (indicating a mutual break in interaction). The infant-gaze-based starting rule was chosen as in rhesus macaques mutual gaze precipitates an opportunity for engagement between mothers and infants, comparable to the opportunity for face-to face interaction in the human sample that was initiated by the researcher’s verbal instructions. To maximize comparability of coding frames across the groups, the same starting rule was applied to the coding of Human mother-infant interactions, commencing as soon as the infant established eye contact with their mother. Human mothers have been found to maintain their gaze directed towards their infant throughout the duration of naturalistic interactions^21,30,159^, and the ending of coding thus coincided with the end of the 3 min of available footage, which is the limit of interactions typically obtained in the age group studied^21^.

Videos were event-coded on a one-second time base, using purpose-built software. Codes included key, mutually exclusive, infant and maternal events described in the literature on mother-infant interactions (which are described in detail below). Infant events were clearly discernible, discrete behaviours with definite onset, thus readily identifiable by the mother in live time (i.e., the infant behaviours were clearly visible for the mother so that she had the chance to see them and react to them). Maternal contingent responses were coded as events occurring within two seconds of each infant event (i.e., starting within two seconds of the infant’s cue). Mean raw frequencies for the main codes reported in the present paper are shown in Supplementary Table S1.

### Infant behaviours (see Table 3)

#### Infant gaze to the mother—duration of time spent by the infant looking at the mother

Since in both humans and macaque interactions, mothers direct their attention almost exclusively to the infant’s face^5,21,22,39^, our coding focused on behaviours involving infant facial movements as well as emissions of sound. Communicative social behaviours described below required the infant to be gazing at their mother’s face, as looking at the interactive partner is one of the characteristics of mother-infant communication in both humans and macaques^39,41,159^, whereas the other groups of infant behaviour were scored independently of gaze direction.

**Table 3:** List of the human and macaque infant behaviours coded.

Infant behaviours	
Human subjects	Rhesus macaques
Proto-communicative mouth gestures Tongue protrusion Mouth opening	Proto-communicative mouth gestures Tongue protrusion Mouth opening
Affiliative gestures (i.e., smiles)	Affiliative gestures (i.e., lip-smacking)
Neutral-Positive vocalisations	Neutral-Positive vocalisations
Non-social mouth movements	Non-social mouth movements
Negative vocalisations	Negative vocalisations
Negative expressions	–
Negative mouth movements	–

#### Communicative social behaviours

(i) proto-communicative mouth gestures: active movements of lips and tongue (e.g., tongue pushed into the bottom lip, moving it forward, or protruded beyond the lips), and of open mouth shaping (e.g., into an ‘O’, or pursed) that appear to be directed at the mother (i.e., infant gazes at mother).
(ii) affiliative/affective behaviours (e.g., smiles in humans, and lip-smacking in monkeys)
(iii) neutral-positive vocalisations, (e.g., cooing in humans, and girns in infant macaques as described in^98^)

We distinguished smiles (human) and lip-smacking (macaque) from the other social expressions because these two behaviours have an important and specific social function in the two groups^39,48,163–165^ as well as a similar affiliative and rewarding value during social interactions^137–139,166^. Similarly, we distinguished infant non-distress vocalizations (i.e., neutral-positive) from the other social behaviours due to their important role in language development and evolution, and because relatively little is known about maternal responses to them in different primate species^97,167,168^, in contrast to the substantial work on responses to infant negative vocalizations in both human and non-human primates^94^.

#### Non-social mouth movements

Mouth movements that appear undirected (e.g., chewing or sucking movements, rolling lips together), or else mouth movements clearly directed to a non-social goal (e.g., rooting to own fist).

#### Negative affect

(i) Vocalisation (e.g., fuss, cry in humans; screams, shrieks, whimpers, coo calls in macaques^79,82,83,98,169^)
(ii) Expression—cry face (only in human subjects);
(iii) Mouth—pout, grimace (only in human subjects).

### Maternal behaviours (see Table 4)

Maternal responses were grouped as follows:

**Table 4:** List of the human and macaque maternal responses coded.

Maternal responses	
Human subjects	Rhesus macaques
Direct mirroring	Direct mirroring
Enriched mirroring	Enriched mirroring
Modified mirroring	Modified mirroring
Marking	Marking
Negative responses	–

#### Mirroring responses

We distinguished the three subcategories we had previously grouped together^23^.

**Figure 3:**
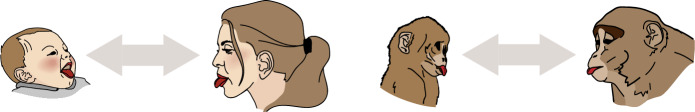
Example of Direct Mirroring (tongue protrusion) in humans (on the left) and rhesus macaque (on the right).

**Figure 4:**
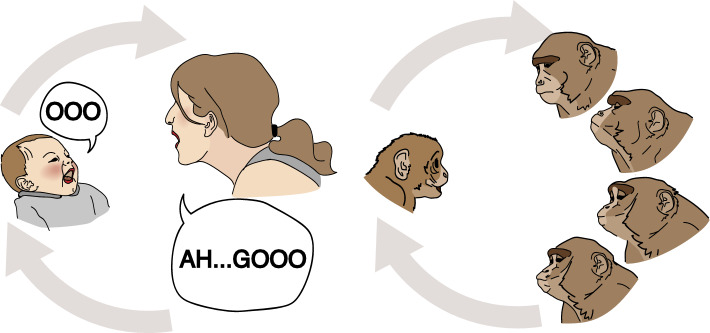
Example of Modified Mirroring (socially meaningful communicative gestures). Positive vocalizations in humans (on the left) and lip-smacking in response to the infant’s open mouth in rhesus macaque (on the right).

**Figure 5:**
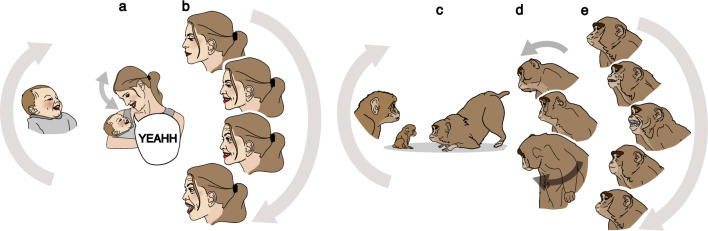
Example of Enriched Mirroring (affiliative gestures). Mother response to infant’s smiles in humans (on the left) and lip-smacking in rhesus macaque (on the right): mother’s smile plus vocal response (**a**) or exaggerated facial expression (**b**) and mother’s lip-smacking plus exaggerated body postures (**c**), head bobbing movements (**d**) or teeth chattering and silent bared teeth (**e**).

(i) Direct mirroring—The mother simply imitates/matches her infant’s behaviour. There is a strong similarity between the infant’s original behaviour and the mother’s response, which matches the form, intensity and affective valence of the infant’s behaviour with no elaboration added (e.g., the mother imitates infant’s mouth opening) (see Fig. 3 for illustration). The code was used for maternal imitations of both single infant behaviours, and multi-component behaviours (e.g., if the infant expressed facial and vocal cues simultaneously and the mother directly imitates both of these components).
(ii) Modified mirroring—The mother’s response shares characteristics of the infant’s behaviour, but alters some elements to create her own version. The mother’s response matches the modality and affective valence of the infant’s behaviour, although not necessarily the form. The modification frequently takes a more prototypical or socially meaningful form (e.g. in humans: the infant vocalises in a non-distinctive manner ( e.g. “ooo”) which the mother mirrors with a more established and defined vocalisation, such as “Ah..Gooo”; or the infant makes a formless tongue protrusion and the mother produces a clear example of ‘sticking out her tongue’; in monkeys: the infant shows a mouth opening or low frequency lip-smacking, and mother responds with a high frequency lip-smacking) (see Fig. 4 for illustration).
(iii) Enriched mirroring—The mother imitates/matches her infant’s behaviour, as in *Direct mirroring*, but also adds some elaboration [e.g. in humans: the mother matches her infant’s smile and also exclaims “aaah” with a happy lilting prosodic contour or laugh, or the mother imitates the infant’s mouth opening and adds the sound “ah”; in monkeys: the infant makes a lip-smacking, and the mother responds with lip-smacking together with head bobbing, head twist, or/and exaggerated postural changes (up-side down head with visual engagement), head approaching with body/head lowering] (see Fig. 5 for illustration).

#### Marking responses

Maternal responses of same valence and intensity as the infant’s behaviour that single out and ‘mark’ an infant behaviour with ‘attention-attracting’ cues, without mirroring it (e.g., in humans: the infant vocalises and the mother responds smiling/nodding/using eyebrow flash and saying “that’s right!”; in monkeys: the infant vocalizes, and the mother responds with lip-smacking).

#### Negating responses (only in human subjects)

(i) Mis-attuned responses (only in human subjects): responses where the affective valence and intensity of the mother’s behaviour is markedly discordant with the infant’s (e.g., the infant shows sign of distress and the mother smiles broadly and laughs; the infant makes a strong positive vocalisation, or gives a strong, ‘joyful’ smile, and the mother responds with a flat, dull, vocalization, or minimal, weak smile).
(ii) Negative, rejecting responses (e.g., the infant makes a cry face and the mother says ‘oh no, don’t do that’ in a harsh tone).

### Coding inter-rater reliability

#### Human subjects

Videos were coded by two researchers, who both independently coded the same 20% of the total sample, including one interaction for each mother-infant dyad. Reliability for infant events was as follows: gaze to mother κ = 0.92; social behaviours κ = 0.92; non-social mouth movements κ = 0.92; negative affect κ = 0.85. Reliability for maternal responses was as follows: total mirroring κ = 0.90; direct mirroring k = 0.80; enriched mirroring k = 0.95; modified mirroring k = 0.91; marking κ = 0.80; negative responses κ = 0.83.

#### Rhesus macaques

Videos were coded by two researchers, who both independently coded the same 21.43% of the total sample. Reliability for infant events was as follows: gaze to mother κ = 0.96; social behaviours κ = 0.97; non-social mouth movements κ > 0.99; negative affect κ = 0.86. Reliability for maternal responses was as follows: total mirroring κ = 0.94; direct mirroring k = 0.85; enriched mirroring k > 0.99; modified mirroring k > 0.99; marking κ = 0.89.

### Data analysis

For each sample, human and macaque, infant events and maternal responses were investigated through Principal Components Analysis (PCA), with Simplimax rotation, and using Parallel Analysis, to determine the number of components to extract.

We used a generalised linear mixed modelling (GLMM) framework to address the study questions. Given the count nature of behavioural variables, two-level random intercept Poisson models were used to analyse the effect of infant age, group, and their interaction on infant and maternal behaviours, using interaction duration as offset, and a dataset including each infant at each age. Three-level random intercept Binomial models were used to compare groups in terms of how mothers distributed their responses across the different infant behaviours, using responded infant behaviours as cases (in a binary form of target behaviour vs. non-target behaviours), and the type of maternal response as predictor, controlling for the base rate of infant behaviours.

For all models, we used Likelihood Ratio Tests (LRT) to assess the effect individual model effects. These LRTs obtained Type 3 tests by comparing models in which only the tested effect was excluded against the full model (full details for each model are provided as Supplementary Information). We used the false discovery rate method for multiple comparisons^170^. A p-value < 0.05 was considered significant.

### Supplementary Information

Supplementary Information 1.

Supplementary Table S1.

Supplementary Table S2.

## Data Availability

The data used for the analysis are available at https://doi.org/10.17638/datacat.liverpool.ac.uk/1653.

## Code availability

The code for the analysis is available at https://doi.org/10.17638/datacat.liverpool.ac.uk/1653.
