# Supplementary material for: Similarities and differences in the functional architecture of mother- infant communication in rhesus macaque and British mother-infant dyads
Source: Sci Rep. 2023 Aug 13;13:13164. doi: 10.1038/s41598-023-39623-3 (PMC10423724; doi:10.1038/s41598-023-39623-3)
Supplement: Supplementary file 1 — Supplementary Information 1. [file 41598_2023_39623_MOESM1_ESM.docx]

**Generalised Linear Mixed Model: Mutual Gaze**

|  | **Mutual Gaze** | | | |
| --- | --- | --- | --- | --- |
| *Predictors* | *Coef.* | *std. Error* | *CI* | *p* |
| (Intercept) | -0.79 | 0.08 | -0.95 – -0.63 | **<0.001** |
| Age | 0.36 | 0.01 | 0.33 – 0.39 | **<0.001** |
| Group (Rhesus Macaques) | -0.01 | 0.16 | -0.32 – 0.29 | 0.932 |
| Age * Group (Rhesus Macaques) | -0.40 | 0.06 | -0.51 – -0.29 | **<0.001** |
| **Random Effects** | | | | |
| σ^2^ | 1.11 | | | |
| τ_00_ _id_ | 0.12 | | | |
| N _id_ | 29 | | | |
| Observations | 115 | | | |

**Generalised Linear Mixed Model: Social Expressiveness**

|  | **Social Expressiveness** | | | |
| --- | --- | --- | --- | --- |
| *Predictors* | *Coef.* | *std. Error* | *CI* | *p* |
| (Intercept) | -0.79 | 0.07 | -0.93 – -0.65 | **<0.001** |
| Age | 0.27 | 0.04 | 0.19 – 0.34 | **<0.001** |
| Group (Rhesus Macaques) | 0.44 | 0.15 | 0.14 – 0.73 | **0.004** |
| Age * Group (Rhesus Macaques) | -0.24 | 0.10 | -0.44 – -0.03 | **0.023** |
| **Random Effects** | | | | |
| σ^2^ | 1.06 | | | |
| τ_00_ _id_ | 0.08 | | | |
| N _id_ | 29 | | | |
| Observations | 83 | | | |

**Generalised Linear Mixed Model: Maternal Responsiveness**

|  | **Maternal Responsiveness** | | | |
| --- | --- | --- | --- | --- |
| *Predictors* | *Coef.* | *std. Error* | *CI* | *p* |
| (Intercept) | -1.22 | 0.11 | -1.44 – -1.00 | **<0.001** |
| Age | 0.14 | 0.04 | 0.05 – 0.22 | **0.002** |
| Group (Rhesus Macaques) | 0.52 | 0.14 | 0.25 – 0.79 | **<0.001** |
| Child Behaviour Rate | -0.00 | 0.01 | -0.02 – 0.01 | 0.540 |
| Age * Group (Rhesus Macaques) | 0.01 | 0.12 | -0.23 – 0.25 | 0.909 |
| **Random Effects** | | | | |
| σ^2^ | 1.43 | | | |
| τ_00_ _id_ | 0.03 | | | |
| N _id_ | 29 | | | |
| Observations | 112 | | | |

**Generalised Linear Mixed Model: Maternal Responsiveness to Infant Social Behaviours**

|  | **Maternal Responses to Social Behaviours** | | | |
| --- | --- | --- | --- | --- |
| *Predictors* | *Log-Mean* | *std. Error* | *CI* | *p* |
| (Intercept) | -0.80 | 0.19 | -1.19 – -0.42 | **<0.001** |
| Age | 0.07 | 0.07 | -0.07 – 0.21 | 0.335 |
| Group (Rhesus Macaques) | 0.46 | 0.18 | 0.10 – 0.82 | **0.013** |
| Child Social Behaviour Ratio | -0.38 | 0.32 | -1.02 – 0.25 | 0.237 |
| Age * Group (Rhesus Macaques) | 0.03 | 0.14 | -0.25 – 0.31 | 0.843 |
| **Random Effects** | | | | |
| σ^2^ | 1.27 | | | |
| τ_00_ _id_ | 0.04 | | | |
| N _id_ | 29 | | | |
| Observations | 79 | | | |

**Generalised Linear Mixed Model: Maternal Mirroring Responses**

|  | **Maternal Mirroring Responses** | | | |
| --- | --- | --- | --- | --- |
| *Predictors* | *Log-Mean* | *std. Error* | *CI* | *p* |
| (Intercept) | -1.36 | 0.26 | -1.88 – -0.85 | **<0.001** |
| Age | 0.17 | 0.10 | -0.03 – 0.37 | 0.101 |
| Group (Rhesus Macaques) | 1.35 | 0.24 | 0.88 – 1.83 | **<0.001** |
| Child Social Behaviour Ratio | -0.98 | 0.43 | -1.82 – -0.14 | **0.022** |
| Age * Group (Rhesus Macaques) | -0.05 | 0.17 | -0.39 – 0.28 | 0.762 |
| **Random Effects** | | | | |
| σ^2^ | 1.80 | | | |
| τ_00_ _id_ | 0.11 | | | |
| N _id_ | 29 | | | |
| Observations | 79 | | | |

**Generalised Linear Mixed Model: Maternal Marking Responses**

|  | **Maternal Marking Responses** | | | |
| --- | --- | --- | --- | --- |
| *Predictors* | *Log-Mean* | *std. Error* | *CI* | *p* |
| (Intercept) | -1.83 | 0.32 | -2.47 – -1.20 | **<0.001** |
| Age | 0.06 | 0.10 | -0.15 – 0.26 | 0.595 |
| Group (Rhesus Macaques) | -0.99 | 0.40 | -1.77 – -0.21 | **0.013** |
| Child Social Behaviour Ratio | 0.16 | 0.54 | -0.90 – 1.22 | 0.766 |
| Age * Group (Rhesus Macaques) | -0.11 | 0.36 | -0.81 – 0.59 | 0.761 |
| **Random Effects** | | | | |
| σ^2^ | 1.99 | | | |
| τ_00_ _id_ | 0.10 | | | |
| N _id_ | 29 | | | |
| Observations | 79 | | | |

**Generalised Linear Mixed Model: Maternal Mirroring Responses to Human Infant Proto-Communicative Mouth Gestures**

|  | **Mirroring Responses to Human Proto-Communicative Mouth Gestures** | | | |
| --- | --- | --- | --- | --- |
| *Predictors* | *Coef.* | *std. Error* | *CI* | *p* |
| (Intercept) | -1.37 | 0.50 | -2.35 – -0.40 | **0.006** |
| Age | -0.52 | 0.25 | -1.01 – -0.03 | **0.038** |
| Enriched Mirroring | 0.48 | 0.47 | -0.44 – 1.40 | 0.309 |
| Modified Mirroring | -3.51 | 0.73 | -4.93 – -2.08 | **<0.001** |
| Infant Behaviour Base Rate | 0.55 | 0.12 | 0.32 – 0.79 | **<0.001** |
| **Random Effects** | | | | |
| σ^2^ | 3.29 | | | |
| τ_00_ _id_ | 0.00 | | | |
| N _id_ | 19 | | | |
| Observations | 157 | | | |

**Generalised Linear Mixed Model: Maternal Mirroring Responses to Human Infant Vocalisations**

|  | **Mirroring Responses to Human Vocalisations** | | | |
| --- | --- | --- | --- | --- |
| *Predictors* | *Coef.* | *std. Error* | *CI* | *p* |
| (Intercept) | -0.54 | 0.49 | -1.50 – 0.43 | 0.279 |
| Age | 0.55 | 0.31 | -0.06 – 1.17 | 0.079 |
| Modified Mirroring | 2.94 | 0.55 | 1.87 – 4.02 | **<0.001** |
| Infant Behaviour Base Rate | -0.16 | 0.09 | -0.33 – 0.01 | 0.072 |
| **Random Effects** | | | | |
| σ^2^ | 3.29 | | | |
| τ_00_ _id_ | 0.00 | | | |
| N _id_ | 17 | | | |
| Observations | 106 | | | |

**Generalised Linear Mixed Model: Maternal Mirroring Responses to Human Infant Communicative Behaviour**

|  | **Mirroring Responses to Human Communicative Behaviour** | | | |
| --- | --- | --- | --- | --- |
| *Predictors* | *Coef.* | *std. Error* | *CI* | *p* |
| (Intercept) | 1.12 | 0.73 | -0.32 – 2.56 | 0.126 |
| Age | -0.38 | 0.32 | -1.01 – 0.25 | 0.240 |
| Enriched Mirroring | -2.25 | 0.65 | -3.53 – -0.97 | **0.001** |
| Modified Mirroring | 2.14 | 1.22 | -0.25 – 4.52 | 0.080 |
| Infant Behaviour Base Rate | 3.34 | 1.28 | 0.83 – 5.85 | **0.009** |
| **Random Effects** | | | | |
| σ^2^ | 3.29 | | | |
| τ_00_ _id_ | 0.58 | | | |
| N _id_ | 19 | | | |
| Observations | 157 | | | |

**Generalised Linear Mixed Model: Maternal Mirroring Responses to Human Infant Smiles**

|  | **Mirroring Responses to Human Vocalisations** | | | |
| --- | --- | --- | --- | --- |
| *Predictors* | *Coef.* | *std. Error* | *CI* | *p* |
| (Intercept) | 18.34 | 0.00 | 18.34 – 18.35 | **<0.001** |
| Age | 7.59 | 0.00 | 7.58 – 7.59 | **<0.001** |
| Enriched Mirroring | 15.26 | 0.00 | 15.25 – 15.26 | **<0.001** |
| Modified Mirroring | -12.77 | 0.00 | -12.77 – -12.76 | **<0.001** |
| Infant Behaviour Base Rate | -20.61 | 0.00 | -20.61 – -20.61 | **<0.001** |
| **Random Effects** | | | | |
| σ^2^ | 3.29 | | | |
| τ_00_ _id_ | 304.91 | | | |
| N _id_ | 19 | | | |
| Observations | 157 | | | |

**Generalised Linear Mixed Model: Maternal Mirroring Responses to Rhesus Macaque Infant Proto-Communicative Mouth Gestures**

|  | **Mirroring Responses to Rhesus Macaque Proto-Communicative Mouth Gestures** | | | |
| --- | --- | --- | --- | --- |
| *Predictors* | *Coef.* | *std. Error* | *CI* | *p* |
| (Intercept) | -0.62 | 1.04 | -2.66 – 1.42 | 0.553 |
| Age | 0.32 | 0.43 | -0.52 – 1.16 | 0.455 |
| Enriched Mirroring | -2.07 | 1.05 | -4.13 – -0.02 | **0.048** |
| Modified Mirroring | 0.48 | 0.99 | -1.47 – 2.42 | 0.631 |
| Infant Behaviour Base Rate | 0.29 | 0.14 | 0.02 – 0.56 | **0.033** |
| **Random Effects** | | | | |
| σ^2^ | 3.29 | | | |
| τ_00_ _id_ | 0.33 | | | |
| N _id_ | 9 | | | |
| Observations | 63 | | | |

**Generalised Linear Mixed Model: Maternal Mirroring Responses to Rhesus Macaque Infant Lip-Smacking**

|  | **Mirroring Responses to Rhesus Macaque Lip-Smacking** | | | |
| --- | --- | --- | --- | --- |
| *Predictors* | *Coef.* | *std. Error* | *CI* | *p* |
| (Intercept) | 0.62 | 1.04 | -1.42 – 2.66 | 0.553 |
| Age | -0.32 | 0.43 | -1.16 – 0.52 | 0.455 |
| Enriched Mirroring | 2.07 | 1.05 | 0.02 – 4.13 | **0.048** |
| Modified Mirroring | -0.48 | 0.99 | -2.42 – 1.47 | 0.631 |
| Infant Behaviour Base Rate | -0.29 | 0.14 | -0.56 – -0.02 | **0.033** |
| **Random Effects** | | | | |
| σ^2^ | 3.29 | | | |
| τ_00_ _id_ | 0.33 | | | |
| N _id_ | 9 | | | |
| Observations | 63 | | | |

**Generalised Linear Mixed Model: Maternal Mirroring Responses to Infant Affiliative Behaviour**

|  | **Mirroring Responses to Affiliative Behaviour** | | | |
| --- | --- | --- | --- | --- |
| *Predictors* | *Log-Odds* | *std. Error* | *CI* | *p* |
| (Intercept) | -1.06 | 0.70 | -2.43 – 0.31 | 0.130 |
| Group (Rhesus Macaques) | 1.37 | 0.88 | -0.35 – 3.09 | 0.118 |
| Enriched Mirroring | 2.31 | 0.67 | 1.00 – 3.62 | **0.001** |
| Modified Mirroring | -2.16 | 1.24 | -4.60 – 0.27 | 0.081 |
| Age | 0.08 | 0.27 | -0.45 – 0.62 | 0.763 |
| Infant Behaviour Base Rate | -3.34 | 1.09 | -5.48 – -1.20 | **0.002** |
| Enriched Mirroring * Group (Rhesus Macaques) | 0.13 | 1.31 | -2.44 – 2.69 | 0.923 |
| Modified Mirroring * Group (Rhesus Macaques) | 1.61 | 1.53 | -1.39 – 4.62 | 0.293 |
| **Random Effects** | | | | |
| σ^2^ | 3.29 | | | |
| τ_00_ _episode:id_ | 0.62 | | | |
| τ_00_ _id_ | 0.19 | | | |
| N _episode_ | 30 | | | |
| N _id_ | 28 | | | |
| Observations | 220 | | | |

**Generalised Linear Mixed Model: Maternal Mirroring Responses to Infant Communicative Behaviour**

|  | **Mirroring Responses to Communicative Behaviour** | | | |
| --- | --- | --- | --- | --- |
| *Predictors* | *Log-Odds* | *std. Error* | *CI* | *p* |
| (Intercept) | 1.06 | 0.70 | -0.31 – 2.43 | 0.130 |
| Group (Rhesus Macaques) | -1.37 | 0.88 | -3.09 – 0.35 | 0.118 |
| Enriched Mirroring | -2.31 | 0.67 | -3.62 – -1.00 | **0.001** |
| Modified Mirroring | 2.16 | 1.24 | -0.27 – 4.60 | 0.081 |
| Age | -0.08 | 0.27 | -0.62 – 0.45 | 0.763 |
| Infant Behaviour Base Rate | 3.34 | 1.09 | 1.20 – 5.48 | **0.002** |
| Enriched Mirroring * Group (Rhesus Macaques) | -0.13 | 1.31 | -2.69 – 2.44 | 0.923 |
| Modified Mirroring * Group (Rhesus Macaques) | -1.61 | 1.53 | -4.62 – 1.39 | 0.293 |
| **Random Effects** | | | | |
| σ^2^ | 3.29 | | | |
| τ_00_ _episode:id_ | 0.62 | | | |
| τ_00_ _id_ | 0.19 | | | |
| N _episode_ | 30 | | | |
| N _id_ | 28 | | | |
| Observations | 220 | | | |
