# Supplementary material for: Similarities and differences in the functional architecture of mother- infant communication in rhesus macaque and British mother-infant dyads
Source: Sci Rep. 2023 Aug 13;13:13164. doi: 10.1038/s41598-023-39623-3 (PMC10423724; doi:10.1038/s41598-023-39623-3)
Supplement: Supplementary file 2 — Supplementary Table S1. [file 41598_2023_39623_MOESM2_ESM.docx]

**Supplementary Table S1. Mean raw frequencies of infant behaviours and maternal responses, according to group and infant age in weeks.**

| **Group** | **Weeks** | **NSMM** | **PCMG** | **S** | **LS** | **PV** | **SB** | **NA** | **GM** | **Mi** | **MiSB** | **Ma** | **MaSB** |
| --- | --- | --- | --- | --- | --- | --- | --- | --- | --- | --- | --- | --- | --- |
| **Humans** | **1** | 5.67 | 0.33 | 0.00 |  | 0.53 | 0.87 | 3.00 | 27.80 | 1.33 | 0.07 | 2.07 | 0.20 |
|  | **3** | 6.93 | 0.93 | 0.13 |  | 1.87 | 2.93 | 3.20 | 42.73 | 0.93 | 0.13 | 3.40 | 0.93 |
|  | **5** | 5.00 | 4.50 | 0.36 |  | 2.29 | 7.14 | 5.50 | 75.86 | 1.71 | 1.14 | 2.93 | 0.64 |
|  | **7** | 6.70 | 10.30 | 0.95 |  | 7.90 | 19.15 | 2.30 | 108.45 | 4.45 | 2.85 | 6.55 | 3.80 |
|  | **9** | 3.58 | 13.00 | 2.26 |  | 9.37 | 24.63 | 1.11 | 133.26 | 5.47 | 4.37 | 6.47 | 4.79 |
| **Rhesus macaques** | **1** | 1.25 | 2.62 |  | 1.25 | 0.00 | 3.88 | 0.25 | 14.83 | 1.83 | 1.62 | 0.54 | 0.33 |
|  | **2** | 1.12 | 4.25 |  | 1.12 | 0.00 | 5.38 | 0.38 | 15.88 | 3.62 | 3.00 | 0.50 | 0.25 |

NSMM: Non-social mouth movements; PCMG: Proto-communicative mouth gestures; S: Smiles; LS: Lip-smacking; PV: Positive Vocalisations; SB: Social Behaviours; NA: Negative Affect; GM: Gaze to Mother; Mi: Mirroring; MiSB: Mirroring of Social Behaviour; Ma: Marking; MaSB: Marking of Social Behaviour
