# Supplementary material for: Similarities and differences in the functional architecture of mother- infant communication in rhesus macaque and British mother-infant dyads
Source: Sci Rep. 2023 Aug 13;13:13164. doi: 10.1038/s41598-023-39623-3 (PMC10423724; doi:10.1038/s41598-023-39623-3)
Supplement: Supplementary file 3 — Supplementary Table S2. [file 41598_2023_39623_MOESM3_ESM.docx]

**Supplementary Table S2. Number and Times of Observations and Face-to-Face Episodes for each infant at each age.**

| ID | Week | Total Number of Observations | Number of Observations with coded face-to-face interactions | Number of face-to-face episodes | Total Recorded Footage | % Codable Footage |
| --- | --- | --- | --- | --- | --- | --- |
| ZJ40 | 1 | 5 | 3 | 9 | 01:49:10 | 1.53 |
| ZJ40 | 2 | 3 | 1 | 2 | 01:10:21 | 0.71 |
| ZJ53 | 1 | 5 | 3 | 8 | 01:36:11 | 3.48 |
| ZJ53 | 2 | 2 | 0 | 0 | 00:21:55 | 0.00 |
| ZJ54 | 1 | 4 | 2 | 5 | 01:38:10 | 2.28 |
| ZJ54 | 2 | 3 | 2 | 4 | 00:51:58 | 1.54 |
| ZJ57 | 1 | 3 | 3 | 3 | 00:56:50 | 0.94 |
| ZJ57 | 2 | 2 | 0 | 0 | 00:25:43 | 0.00 |
| ZJ59 | 1 | 2 | 2 | 2 | 00:36:41 | 0.55 |
| ZJ59 | 2 | 1 | 0 | 0 | 00:17:30 | 0.00 |
| ZK10 | 1 | 5 | 2 | 6 | 01:32:55 | 2.17 |
| ZK10 | 2 | 3 | 1 | 5 | 01:02:29 | 1.89 |
| ZK11 | 1 | 6 | 2 | 2 | 01:24:58 | 0.41 |
| ZK11 | 2 | 4 | 1 | 1 | 00:34:11 | 1.27 |
| ZK14 | 1 | 4 | 4 | 7 | 01:02:52 | 1.72 |
| ZK14 | 2 | 4 | 1 | 1 | 00:48:49 | 0.10 |
| ZK26 | 1 | 4 | 3 | 6 | 01:03:01 | 1.48 |
| ZK26 | 2 | 3 | 2 | 5 | 00:28:05 | 4.09 |
